# Detection of Double-Compressed Videos Using Descriptors of Video Encoders

**DOI:** 10.3390/s22239291

**Published:** 2022-11-29

**Authors:** Yun Gu Lee, Gihyun Na, Junseok Byun

**Affiliations:** 1 School of Software, Kwangwoon University, Seoul 01897, Republic of Korea; 2 Digital Analysis Division, National Forensic Service, Gangwondo 26460, Republic of Korea

**Keywords:** video forgery detection, video tampering detection, double compression detection, digital forensics

## Abstract

In digital forensics, video becomes important evidence in an accident or a crime. However, video editing programs are easily available in the market, and even non-experts can delete or modify a section of an evidence video that contains adverse evidence. The tampered video is compressed again and stored. Therefore, detecting a double-compressed video is one of the important methods in the field of digital video tampering detection. In this paper, we present a new approach to detecting a double-compressed video using the proposed descriptors of video encoders. The implementation of real-time video encoders is so complex that manufacturers should develop hardware video encoders considering a trade-off between complexity and performance. According to our observation, hardware video encoders practically do not use all possible encoding modes defined in the video coding standard but only a subset of the encoding modes. The proposed method defines this subset of encoding modes as the descriptor of the video encoder. If a video is double-compressed, the descriptor of the double-compressed video is changed to the descriptor of the video encoder used for double-compression. Therefore, the proposed method detects the double-compressed video by checking whether the descriptor of the test video is changed or not. In our experiments, we show descriptors of various H.264 and High-Efficiency Video Coding (HEVC) video encoders and demonstrate that our proposed method successfully detects double-compressed videos in most cases.

## 1. Introduction

The price of digital imaging systems today has become considerably low, and the use of devices equipped with cameras has become a fact of daily life. Numerous surveillance cameras on roads and streets are constantly recording our surroundings, and when an accident or a crime occurs, video becomes important evidence for digital forensics [1,2]. For example, a video recorded by a car’s digital video recorder (DVR) camera (or black box camera) can be used as important evidence in the case of a car accident. Digital forensics is a forensic science that encompasses all types of digital media devices and digital technologies [3,4]. Delp defines digital forensics as scientific techniques for the preservation, collection, validation, identification, analysis, interpretation, documentation, and presentation of digital media evidence acquired from digital devices [5]. On the other hand, video editing programs are becoming popular, and even non-professional users can easily use such programs to delete or modify a section of an evidence video that contains adverse evidence. Therefore, detecting a forgery video plays a key role in digital forensics.

**Figure 1:**
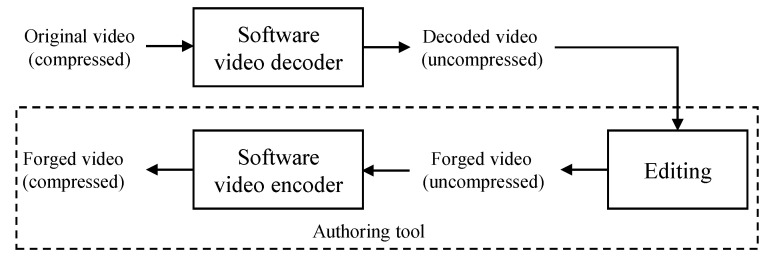
Procedure of video forgery by double compression.

A video basically consists of a sequence of images (or frames). The forgery video can be detected by applying image tampering detection methods [6,7,8] to each frame of the video. However, the performance of this approach was not satisfied due to many reasons [9]. Therefore, many algorithms have been proposed to detect forgery videos. Sitara [9] classified video tampering detection methods into three types: double compression detection, video inter-frame forgery detection, and region tampering detection. In video inter-frame forgery, a target video is tampering in the temporal domain. Typical methods are frame removal, frame duplication, and frame insertion. Since this type tampers the target video in the temporal domain, detection methods usually analyze motion, prediction error, and residuals in P frames [10,11,12,13]. In region tampering, an attacker copies a small region of a frame and pastes it at another frame. Chen [14] proposed a method to detect region tampering using features from motion residuals. Lin’s method utilizes temporal copy-and-paste and exemplar-based texture synthesis to detect tampered videos [15]. Su [16] utilized the difference between the current and a non-tampered reference frame to detect dynamic foreground removal from static background video. The last type of video tampering detection is double compression detection. Figure 1 represents a procedure of video forgery using double compression. The original video (compressed) is decompressed first. Then, an attacker modifies the decoded video (uncompressed) using editing software according to his or her wish. Finally, the forged video is compressed using a software video encoder again. To detect a double-compressed video, Wang [17] proposed a method using specific static and temporal statistical perturbations of a double-compressed Moving Picture Experts Group (MPEG) video. The double quantization effect is used in [18] to detect double MPEG compression. Markov-based features are proposed to detect double compression artifacts in [19]. A method to detect double Advanced Video Coding (AVC)/HEVC [20,21] encoding was proposed under the assumption that the former compression has a lower quality than the latter compression [22]. In [23], the probability distribution of quantized non-zero AC coefficients is utilized as features to detect double-compressed video. He et al. [24] proposed a method to detect double compression based on local motion vector field analysis in static-background videos. Bestagini [25] proposed a method to identify the codec and the size of a group of pictures that are used in the first coding step and analyze double-compressed videos. Based on quality degradation mechanism analysis, Jiang et al. [26] proposed a method to detect double compression with the same coding parameters. Recently, Li [27] proposed a semi-supervised learning method to detect double-compressed video using Gaussian density-based one-class classifiers. Mahfoudi proposed the statistical H.264 double-compression detection method based on discrete cosine transform (DCT) coefficients [28]. A motion-adaptive algorithm is proposed to detect HEVC double compression with the same coding parameters [29]. Since video forgery is normally performed on uncompressed domains, the process of video tampering consists of decoding the compressed video, editing the uncompressed video, and compressing the edited video again [27]. Therefore, double compression detection is generally an effective method of video forensics [14,27,30]. Hence, our study focuses on double compression detection.

This paper presents a novel method to detect a double-compressed video under conditions that are slightly limited but frequently occur in practice. There are two assumptions in our study. The first assumption is that the model name of a camera that took a test video is known. Here, the test video denotes the video to be checked for tampering. Since the test video is submitted as evidence of a crime, the model name of the camera that took the test video is generally known. The second assumption is that an attacker is not enough of a video coding expert to develop his (or her) own software video encoder. Therefore, when compressing a forged uncompressed video, the attacker utilizes a software video encoder available either publicly or on the market. In other words, the software video encoder shown in Figure 1 should be available publicly or on the market. The main idea behind the proposed method is that video encoders usually utilize a subset of encoding modes among all possible modes in a trade-off between complexity and performance. The proposed method defines this subset of encoding modes as a descriptor for each video encoder. If the test video is double-compressed for forgery, the descriptor of the double-compressed video should belong to the descriptor of the software video encoder used for the last compression, not the descriptor of the camera model from which the test video was taken. Therefore, the proposed method detects the double-compressed video by comparing the descriptor of the camera model with the descriptor of the test video.

The contributions and novel parts of our study are as follows. First, this paper introduces a new approach in the field of detecting a double-compressed video. While most existing methods analyze only the test video itself, the proposed algorithm considers the characteristics of the hardware video encoder that took the test video. This approach is one of the novel parts of our study. Second, the proposed method can complement the existing method to further improve detection accuracy. For example, the proposed method first checks a test video to see whether it is tampered with or not. If the proposed method fails to detect anything, the existing methods can be applied to further examine the test video. Finally, if the proposed method decides a test video is a forgery video, the probability of a wrong decision is extremely low, which can be used for strong evidence in a crime. There is no risk that an unforged video is determined to be a forgery video.

The rest of the paper is organized as follows. Section 2 illustrates how video encoders utilize a subset of encoding modes among all possible modes for a trade-off. Section 3 introduces the H.264 and HEVC descriptor and proposes a method for detecting a double-compressed video. In Section 4, we show experimental results. Finally, we conclude our work in Section 5.

## 2. Characteristics of Hardware Video Encoder

### 2.1. Encoder Complexity

A video encoder for international video coding standards, such as using H.264 [20] and HEVC [21], is usually much more complex than the corresponding video decoder [31,32]. The video encoder should choose the best prediction modes from possible candidates, which requires high computation. As the number of modes (or options) to choose increases, the complexity of the video encoder increases. Let us consider the encoder complexity in brief in terms of block sizes and prediction modes. In H.264, a macroblock (MB) of a size of 16×16 is encoded in an intra mode or an inter mode. The intra mode supports two types of block sizes: 16×16 blocks or four 4×4 blocks. There are nine intra prediction modes in the 4×4 block and four intra prediction modes in the 16×16 block. The detailed prediction modes are given in [20]. The video encoder should decide the block size of the intra block, whether it is predicted as a 16×16 block or four or a 4×4 block. It also predicts the intra prediction mode for each sub-block. In the inter mode, the best matching block for an MB is found within previously reconstructed frames, which is called motion estimation and compensation. H.264 supports a block partition technique that divides the MB into sub-blocks to improve motion compensation performance. The MB is first partitioned into one of 16×16, 16×8, 8×16, and 8×8. For the 8×8 partition case, each 8×8 block can be further partitioned into 8×8, 8×4, 4×8, or 4×4 blocks. The video encoder should estimate the block partition size for each MB. HEVC is an international video coding standard that supports more flexible prediction modes than H.264. While the basic coding unit of H.264 is an MB of size 16×16, the basic processing unit of the HEVC is a coding tree unit (CTU) [21] whose size is variable: 64×64, 32×32, 16×16, and 8×8. Further, the number of intra prediction modes in HEVC is 35, which is much more than those of H.264. Therefore, the complexity of HEVC is higher than that of H.264.

There are two approaches to the implementation of a video encoder: software and hardware. Software-based video encoders are inherently very flexible in modifying and upgrading algorithms. Further, there is no limitation on the size of input videos. As the size increases, the processing time increases only. This flexibility makes the software-based encoder adaptable to various types of applications. As the software-based encoder does not guarantee real-time processing, it is suitable for offline applications. Meanwhile, since the processing blocks in a hardware-based encoder run in parallel, its processing speed (or throughput) is generally faster than that of a software-based encoder. However, the hardware video encoder cannot process a video larger than the designed hardware specification due to limitations of memory size, operation frequency, etc. Once the video encoder is implemented in an application-specific integrated circuit (ASIC), it is very difficult to modify or upgrade the encoding algorithms. Hence, hardware-based video encoders are suitable for consumer electronics where real-time encoding is an essential feature.

### 2.2. Implementation of Video Encoder in Hardware

This paper proposes a method to detect a video forged from a video shot with a camera, such as a car DVR (or a black box). Here, the camera encodes input frames using a hardware video encoder. Hence, this subsection examines the hardware implementation of video encoders.

To achieve high coding performance, video encoders generally need to examine all possible prediction modes and choose the best mode among the candidates. For example, HEVC video encoders need to examine 35 intra-prediction candidates and choose the best one among the 35 candidates. Recent video coding standards are so complex that even hardware-based video encoders are not practical to carefully check and evaluate all possible prediction modes in real-time. Therefore, many researchers have proposed new hardware architectures with novel fast algorithms to efficiently reduce the processing speed and implementation cost while maintaining the coding efficiency. Video encoders need to determine many parameters, such as block size, block partition, intra prediction mode, and so on. Top-tier hardware vendors can develop all new advanced algorithms that run in real-time for high-performance video encoders. However, since the development of the new advanced algorithms increases the development cost, other hardware vendors may find it difficult to develop all new advanced algorithms to determine encoding modes. These hardware vendors may need to consider a trade-off between the development cost and the encoder performance. Therefore, some hardware vendors often consider simple and straightforward methods to efficiently reduce the development cost while the performance degradation is not significant.

One of the simple and straightforward methods is to use only a subset of encoding modes among all possible ones. Since three intra predictions of modes 0 (vertical), 1 (horizontal), and 2 (DC) for 4×4 block are statistically dominant in most video sequences; hardware vendors may develop a video encoder that uses only the three intra prediction modes among the nine intra prediction modes in 4×4 blocks. This encoder does not include any hardware block for processing or predicting the six intra prediction modes. Although this simple approach may degrade the coding performance for some videos, it significantly reduces the development cost, hardware complexity, and processing speed. According to our observations, many hardware manufacturers frequently adopt this simple way of implementation. This proposed method utilizes this feature to detect a tampered video. In the experimental results, we will show real examples of this simple implementation.

For a video decoder to comply with the video coding standard, the decoder should have the capability to decode all encoding modes defined in the standard. On the other hand, if the bitstream generated from a video encoder is decodable by the standard decoder, the video encoder is considered to generate the bitstream that conforms to the video coding standard. Hence, the video encoder can comply with the standard even if only some of encoding modes defined by the standard are implemented. For example, an H.264 video encoder that uses only three intra prediction modes for the 4×4 intra block complies with the video coding standard.

## 3. Detection of a Double-Compressed Video

### 3.1. Descriptor of a Hardware Video Encoder

#### 3.1.1. Descriptor Structure

As mentioned in the previous section, some hardware video encoders support only a subset of encoding modes among all possible ones. Which encoding modes are included in the subset can vary from video encoder to video encoder. Hence, the subset can be used as the descriptor of a video encoder. Let us first examine the descriptor of an H.264 video encoder. There are many encoding modes to be determined by the H.264 encoder. The proposed descriptor includes the intra prediction modes and inter block partition. The profile, level, and group of pictures (GOP) are also included in the descriptor. Table 1 illustrates an example of the H.264 video encoder’s descriptor. In this example, the video encoder supports the baseline profile with level 3.1. A plane intra prediction mode (or mode 3) is not used in intra 16×16. The encoder considers only three prediction modes of 0, 1, and 2 for intra 4×4. The 8×8 block in the inter block is not decomposed further. This video encoder supports limited encoding modes compared to the full features of the baseline profile.

The proposed HEVC descriptor basically includes some parameters from the video parameter set (VPS), sequence parameter set (SPS), and picture parameter set (PPS), such as CTU size, minimum coding unit (CU) size, and GOP size. The descriptor also includes the intra prediction modes and block decomposition types, such as the descriptor of the H.264 video encoder. Since the block partitioning structure in the HEVC standard is very complex, the definition of the HEVC descriptor is not simple. Let us review its block partitioning structure in brief. As explained in the previous subsection, the basic processing unit of the HEVC is CTU, whose size is from 8×8 up to 64×64. The CTU is recursively partitioned into multiple CUs based on a quadtree structure. The minimum size of the CU is specified in the SPS. For example, a CTU of the size 32×32 can be decomposed into CUs of the size 32×32, 16×16, and 8×8. Each CU in HEVC is categorized into one of three types: skipped CU, inter coded CU, and intra coded CU. Here, inter and intra coded CUs are further split into multiple prediction units (PU). HEVC supports two types of PUs for the intra CU: 2N×2N and N×N. For the inter CU, there are eight types of PUs: 2N×2N, N×N, 2N×N, N×2N, 2N×nU, 2N×nD, nL×2N, and uR×2N. For example, a CU of the size 64×64 can be split into 64×64 and 32×32 PUs for intra CU and 64×64, 64×32, 32×64, 64×16, 64×48, 16×64, 48×64, and 32×32 PUs for inter CU. If the size of a CU is 32×32, the CU can be partitioned into 32×32 and 16×16 PUs for intra CU and 32×32, 32×16, 16×32, 32×8, 32×24, 8×32, 24×32, and 16×16 PUs for inter CU. Hence, the structure of the HEVC descriptor will be very complex, like the block partitioning structure of the HEVC.

It is not easy to organize the proposed HEVC descriptor into a single table due to the complex coding tree structure, which consists of several tables. The proposed method constructs the descriptor according to the size and type of the CU that is a leaf node of the coding tree rather than the coding tree structure of the CTU. Table 2 represents an example of the HEVC encoder’s descriptor according to the CU sizes and types. Splitting CU into four equal-size PUs (or N×N) is conceptually similar to four equal-size CU partitions with 2N×2N. For example, four 16×16 PUs for 32×32 CU is like four 16×16 PUs for four 16×16 CUs. Hence, HEVC allows N×N PUs only if the CU size is equal to the minimum CU size. The HEVC descriptor in Table 2 requires four additional tables to show the intra prediction modes: three 2N×2N intra prediction mode tables for 32×32 CU, 16×16 CU, and 8×8 CU and one intra N×N prediction mode table for 4×4 CU. Table 3 shows an example of intra 2N×2N prediction modes for 32×32 CU. The HEVC descriptor also includes the block partitioning information for inter coded CUs. Each inter coded CU in Table 2 has a corresponding table to describe the block partitioning information. Table 4 represents an example of the block partitioning information of a 32×32 inter CU. In this example, the HEVC encoder utilizes only 2N×2N PU.

#### 3.1.2. Prediction of Encoder’s Descriptor

Hardware video encoder manufacturers usually provide the basic specifications of video encoders, such as the profile and level, which are some of the components of the proposed descriptor. Even if the manufacturers do not provide the specification, these values for the descriptor are directly readable from VPS, SPS, and PPS. However, most values of the descriptors in Table 1, Table 2, Table 3 and Table 4 are closely related to algorithms adopted by the video encoder, and the algorithms are rarely public. For example, intra prediction and inter block partition algorithms are not public. Once a video encoder is implemented in the hardware, it is not possible to figure out how algorithms work in the encoder. Therefore, it is not possible to directly find the values in the descriptor through algorithms. We cannot see the internal processing of a video encoder, but we can generate tons of video clips with the video encoder. Therefore, in this paper, we present an indirect method by statistically analyzing the video clips and predicting the values of the proposed descriptor.

The descriptor analyzer monitors the process of decoding video clips. Whenever a mode in the proposed descriptor is found in the video clip, the analyzer increases the variable to record the number of how many times the corresponding mode is used in the video clip. For example, let us assume that an H.264 decoder processes an MB in a video clip. If the MB is an intra 16×16 and its prediction mode is 0 (DC), the analyzer increases the variable for an intra prediction mode 0 of intra 16×16 block by 1. The number of MBs in a single frame with a full high-definition (HD) resolution is 8160. There are $2.448\times 10^{6}$ MBs in a full HD video with 30 frames per second (fps) if the running time of the video is 10 s. Hence, the number of MBs (or CTU) from video clips is so high that the probability of not selecting the mode used in the video encoder is very low. If the number of videos for analysis is insufficient, we can shoot new videos and analyze them. Consequently, the descriptor analyzer can statistically figure out which modes are used in the encoder by decoding a video clip.

### 3.2. Tampered Video Detection

**Figure 2:**
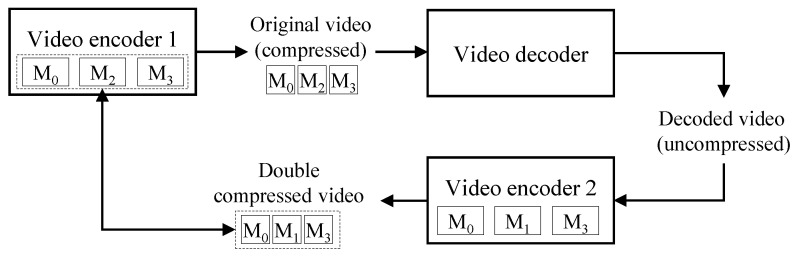
Key idea of the proposed algorithm.

Figure 2 depicts the key idea of the proposed method. Video encoder 1 supports encoding modes of $M_{0}$, $M_{2}$, and $M_{3}$. Therefore, the descriptor of a video compressed by video encoder 1 also has encoding modes of $M_{0}$, $M_{2}$, and $M_{3}$, which are the characteristics of video encoder 1. In the process of decoding the compressed video, the uncompressed video loses the characteristics of video encoder 1. A double-compressed video has the characteristics of video encoder 2, whose descriptor includes $M_{0}$, $M_{1}$, and $M_{3}$. The descriptor of the double-compressed video is different from the descriptor of video encoder 1. Therefore, the proposed method compares the descriptor of a test video with the descriptor of the video encoder used in the first compression. If the test video is a double-compressed video, its descriptor may be different from that of the video encoder used in the first compression. The details will be given in the following sections.

#### 3.2.1. Structure of the Proposed Detector

Let us assume that a test video (or an evidence video) is submitted to check whether the video is forged or not. Conventional methods usually focus on the test video itself. They carefully analyze the test video to find out tiny changes due to double compression. Meanwhile, the proposed method takes a different approach from the previous methods. It does not analyze only the test video but compares the test video with other videos shot by the same video encoder from the viewpoint of the proposed descriptor. Since the test video is submitted as evidence of a crime, the model name of the camera (or video encoder) that took the test video is generally known. We can generate new videos using the same camera if necessary. Hence, the test video can be compared to other videos shot with the same camera.

**Figure 3:**
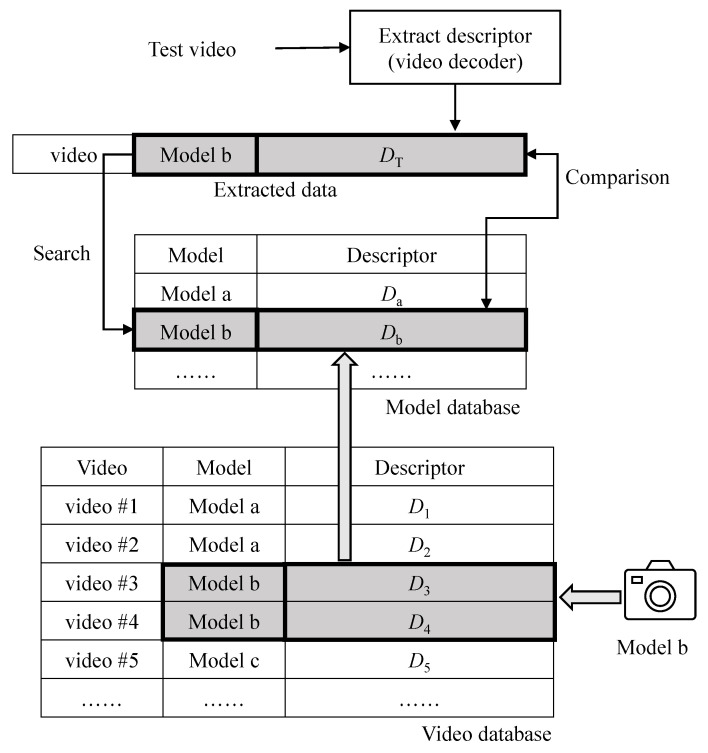
Proposed detection system.

Figure 3 illustrates the proposed detection system. The first step of the proposed system is to decode a test video and extract its descriptor or $D_{T}$ according to the method described in Section 3.1.2. In digital forensics, when the test video (or evidence video) is submitted, the model name of the camera that the video evidence was taken with is usually provided. In the figure, the model name for the test video is *Model b*. Then, the proposed method searches the descriptor of the corresponding model (*Model b*) in the model database. If the descriptor for the corresponding camera model is not in the model database, new videos are taken using a camera of *Model b*. Then, the proposed method predicts the descriptors of the new videos and stores them in the video database. In the figure, two videos, #3 and #4, were taken, and their descriptors are $D_{3}$ and $D_{4}$, respectively. The descriptors of the new videos are merged to generate the video encoder’s descriptor (or $D_{b}$) in the model database. Here, the numbers to indicate how many times each mode is used are simply added for the encoder’s descriptor. Finally, the proposed method compares the test video’s descriptor ($D_{T}$) to that of the video encoder’s descriptor ($D_{b}$) to determine whether the test video is tampered with or not.

#### 3.2.2. Comparison of Descriptors

In Figure 3, the descriptors predicted from videos taken with the same camera are merged to generate the descriptor in the model database. The number of times each mode is used in a video is recorded in a descriptor in the video database. The proposed method simply adds the number of descriptors in the video database. If a particular mode is implemented in the video hardware, the number of times corresponding to the mode will be greater than zero. When comparing two descriptors, the proposed scheme compares whether a specific mode is implemented or not. Therefore, the scheme does not directly compare the number of two descriptors but rather checks whether each number is greater than zero. In Table 5, the numbers for $M_{0}$, $M_{1}$, and $M_{2}$ in videos #1 and #2 are greater than zero, and the numbers for $M_{3}$ and $M_{4}$ are zero. Hence, the descriptors of two videos of #1 and #2 are consistent with each other. However, the numbers of $M_{0}$, $M_{1}$, $M_{2}$, $M_{3}$, and $M_{4}$ in video #3 are 15249, 292342, 0, 125242, and 8754, respectively. Only the number of $M_{2}$ is zero, and the others are non-zero. Hence, the descriptor of video #3 is not consistent with the descriptors of video #1 or #2.

There is an important issue. We mentioned in Section 3.1.2 that the method can statistically predict the video encoder’s descriptor if the number of videos for analysis is sufficient. Since we know the camera model used to shoot the evidence video, we can take as many videos as we need, and the above assumption is valid for predicting the descriptors of video encoders. The issue is that the test video’s descriptor is predicted from a single video. Is the descriptor from the single video comparable to the video encoder’s descriptor predicted from multiple videos? It depends on the statistical situation. Table 6 shows an example of the desirable case in H.264. Videos #1 and #2 are shot by the same camera model. The running times of the two videos are 60 and 20 s, respectively. In this example, the video encoder determines 1.19% and 5.59% of the total MBs as 4×4 intra prediction mode. The total numbers of MBs for 4×4 intra prediction mode are 174,639 and 273,562, respectively. Hence, the number of MBs is statistically enough to determine whether the video encoder includes the hardware logic for the corresponding mode. In this case, the descriptor from the single test video is identical to the video encoder’s descriptor predicted from multiple videos. Now let us consider another example in Table 7. This table represents an example of an undesirable case in HEVC. Videos #1 and #2 are shot by the same camera model. The running times of the two videos are 5 and 5 s, respectively. The total number of CUs for 2N×2N intra prediction of 32×32 CU are 6,851 and 70,871, respectively, which are 2.2% and 23%. While the number of H.264 intra prediction types is 9, the number of HEVC intra prediction modes is 35, which is much larger than H.264’s. Further, in this example, most intra prediction modes belong to DC and planar modes (more than 80%), rather than angular prediction. Therefore, the numbers that belong to angular prediction are not statistically enough to determine whether the video encoder includes the hardware logic for the mode. Mode 4 was used in video #2 but not in video #1. In this situation, the descriptor from the single test video will not be identical to the video encoder’s descriptor, even if they were shot by the same camera model. On the other hand, the descriptor in the model database is a super-set of all descriptors of videos taken with the same camera model. Hence, videos #1 and #2 are subsets of the descriptor in the model DB, as shown in the table.

When comparing the descriptor of the test video to the descriptor of the video encoder, there are three types of comparison results. Table 8 represents an example of the three types. The first case ($case_{1}$) is when the descriptor of a video encoder in a model database ($D_{b}$) is identical to the descriptor of the test video #1 ($D_{T}^{1}$), which is $D_{b}=D_{T}^{1}$. In the second one ($case_{2}$), the descriptor of the test video ($D_{T}^{2})$ is included in the descriptor of the video encoder ($D_{b}$). In other words, the test video’s descriptor is a subset of the video encoder’s ($D_{T}^{2}\subset D_{b}$). The last one ($case_{3}$) is a case where the descriptors of the test video and the video encoder are not related to each other. In the table, there is no relation between $D_{b}$ and $D_{T}^{3}$.

**Figure 4:**
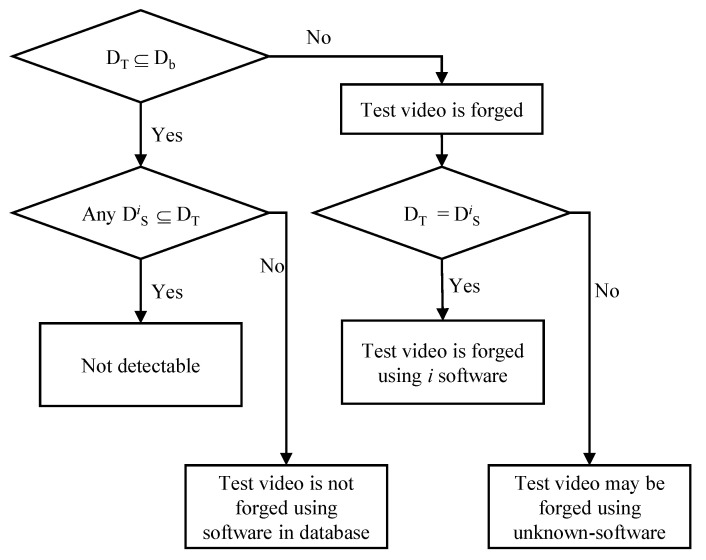
Proposed decision rule. $D_{T}$, $D_{b}$, and $D_{S}^{i}$ are descriptors of the test video, the video encoder, and the *i* software video encoder, respectively.

If the test video is not double compressed, the descriptor of the test video should be consistent with or included in the descriptor of the video encoder used to shoot the test video ($D_{T}\subseteq D_{b}$). If not ($D_{T}⊈D_{b}$), the test video is considered to be forged from the original one. An attacker usually compresses the tampered video (uncompressed) using a software video encoder, such as FFmpeg [33]-based software, Adobe Premier, etc. We can analyze various types of software video encoders, predict their descriptors, and store their descriptors in the model database in advance. When the test video is determined to be forged, we may find which software video encoder is used in forging the test video by comparing the test video’s descriptor with descriptors from various software video encoders in the model database, as shown in Figure 4. In this case, the descriptor of the test video ($D_{T}$) is compared to the descriptors of the software video encoders in the model database ($D_{S}^{i}$). If $D_{T}$ is a subset of $D_{S}^{i}$ ($D_{T}\subseteq D_{S}^{i}$), the test video may be forged using the *i* software.

Now let us consider a case that the descriptor of the test video is included in or consistent with the video encoder’s descriptor in the model database ($D_{T}\subseteq D_{b}$). In this case, we compare the test video’s descriptor with all descriptors of the software encoder in the database. If no software encoder’s descriptor in the model database is a subset of the test video’s descriptor, we conclude that the test video is not double compressed using software video encoders used in to build the database. If the model database includes descriptors of most authoring tools used for the forgery of videos and no matching descriptor is found, it is highly probable that the test video is not double compressed. Unfortunately, if any $D_{S}^{i}\subseteq D_{T}$, we cannot be sure whether the test video is the original one taken with the same camera model used to build the model database. There is a possibility that the test video is double compressed using the software encoder that has the same descriptor as the original camera model. Hence, the proposed method categorizes this case as *Not detectable*.

### 3.3. Refinement of H.264 Descriptors

In the process of decoding videos and extracting H.264 descriptors, we found a peculiar result, as shown in Table 9. Although a particular mode is implemented in some hardware, it is rarely used. In the table, the probability of choosing the 16×16 intra prediction mode 3 is less than 0.01%, and the probability for the remaining three modes is 99.99%. Since the mode decision algorithm inside the chip (hardware video encoder) is not public, the exact reason cannot be analyzed, but it is assumed for the following reason. HEVC video encoder solutions are still very expensive, so only hardware vendors with advanced technology can develop them. Meanwhile, H.264 video encoder solutions are popular nowadays, so even low- or mid-range hardware vendors can develop them. It is expected that the low- or mid-range vendors might develop immature algorithms, and a particular mode is rarely chosen. Although the particular mode is implemented in these hardware video encoders, since the mode is rarely chosen, our method considers that the rarely chosen mode is the ‘unused mode’. In the descriptor, the mode will be marked as ‘×’. If the probability of choosing a particular mode is less than 0.01%, the proposed method considers the corresponding mode as the ‘unused mode’.

## 4. Experimental Results

Versatile video coding (VVC) [34] is a new generation of the international video coding standard that was developed by the ITU-T and the ISO/IEC. Unfortunately, VVC hardware encoders are not available in the consumer market yet. The predecessors, such as H.264 and HEVC, are still popular, and the most available hardware video encoders in the market belong to either H.264 or HEVC. In recent years, the low cost of the H.264 video encoder has led to it being widely adopted in low-end and mid-end devices. Since the HEVC video encoder is relatively expensive, high-end devices are often equipped with the HEVC video encoder. Accordingly, we built the database and performed experiments for H.264 and HEVC. As described in Section 3.2.2, the proposed method does not directly compare the number of times in the two descriptors but compares whether each mode is used or not. Hence, the tables in the following sections will not indicate the number of times each mode is used but indicates whether each mode is used with O or ×.

### 4.1. Descriptors of HEVC Hardware Video Encoder

In experiments, eight models of car DVR (or black box) cameras, four models of smartphone cameras, and one GoPro camera were used to evaluate the proposed method. We took many videos using the above cameras to build the model database. The total running time of videos shot by each camera model was more than 5 min (9000 frames). Video resolutions from the car DVR cameras are 2560×1440. The resolution of videos shot by the GoPro is 3840×2160. The remaining video’s resolution is 1920×1080.

Section 2.2 mentioned that hardware video encoders often adopt simple and straightforward methods of using only a subset of the encoding modes among all possible modes. This subset, indicating whether a particular encoding mode is implemented or not, becomes a descriptor. Since the structure of the HEVC descriptor is very complex, as described in Section 3.1.1, it is not appropriate in this paper to show the full descriptor for all camera models. Further, it is very difficult to compare huge sizes of descriptors from different camera models one by one. Hence, this paper summarizes the descriptors in terms of intra 2N×2N prediction, intra N×N prediction, and inter block partitioning.

Table 10 represents examples of intra 2N×2N prediction modes of 32×32, 16×16, and 8×8 CUs in HEVC encoder’s descriptor. The CTU size of all camera models used in the experiments was equal to 32×32. The minimum CU size of one of the camera models is 16×16, and the minimum CU size of the other camera models is 8×8. Since DC and planar prediction modes in intra 2N×2N prediction (mode 0 and 1) are very important, these modes are commonly utilized in all CUs of 32×32, 16×16, and 8×8 in all camera models. Camera model 1 uses all angular prediction modes in only 8×8 CU. It considers subsets of angular prediction modes for 32×32 and 16×16 CUs. Mode2, mode10, mode18, mode26, and mode34 are used in 32×32 CU. Only angular prediction modes with even indices are considered in 16×16 CU. Camera model 2 implemented a subset of intra prediction modes in 32×32 CU and a full set of intra prediction modes in 16×16 and 8×8 CUs. Intra 2N×2N prediction descriptors for Camera models 2 and 3 are identical except for mode 34 in 32×32 CU. Although we performed a lot of experiments, videos shot with Camera model 4 did not include mode 33, regardless of the CU size. Camera model 5 supports the full intra 2N×2N prediction modes. Camera model 6, whose minimum CU size is 16×16, also supports the full intra 2N×2N prediction modes. Since the hardware logic to perform intra 2N×2N predicted for a large block, such as 32×32 and 16×16, is expensive, it is expected that the implementation of a limited number of intra 2N×2N prediction modes is preferred in some hardware video encoders.

Four 8×8 PUs for 16×16 CU are similar to four 8×8 PUs for four 8×8 CUs. Hence, HEVC allows N×N PUs only for the minimum CU size. Table 11 shows examples of the intra N×N prediction mode of 8×8 CU in the HEVC encoder’s descriptor. Camera model a did not implement the hardware logic for intra N×N prediction modes in 8×8 CU. Camera model b supports DC and planar prediction modes as well as intra prediction mode with odd indices. Camera models c and *d* do not consider modes 33 and 34, respectively. In fact, camera model c in Table 11 and camera model 4 in Table 10 are the same camera model. Camera model e supports a full set of intra N×N prediction modes for 8×8 CU.

Table 12 shows examples of block partitioning of inter coded CU in HEVC encoder’s descriptor. HEVC has eight types of PUs for the inter CU: 2N×2N, N×N, 2N×N, N×2N, 2N×nU, 2N×nD, nL×2N, and uR×2N PUs. Each type of PU is represented in the table as 0, 1, 2, 3, 4, 5, 6, and 7. For example, index 4 corresponds to 2N×nU. Camera model *a* considers only 2N×2N inter predictions in all CU sizes. Camera model *b* utilizes 2N×2N, N×N, and 2N×N inter block partitions in 32×32 and 16×16 CU sizes, but it adopts only 2N×2N inter prediction in the 8×8 CU size. Since four N×N PUs (index 3) for 32×32 (or 16×16) CUs are identical to four 2N×2N PUs (index 0) for four 16×16 (or 8×8) CUs, camera model *c* can be considered to support all block partitions in 32×32 and 16×16 CU sizes. It supports three block partitions in 8×8 CU. The minimum CU size of camera model d is 16×16. This camera model supports three partitions in 32×32 CU and four partitions in 16×16 CU.

As described above, the hardware video encoders used in our experiments support a subset of encoding modes rather than the full set of encoding modes. According to our analysis, the descriptors of camera models used in our experiments are unique to each other. However, there are many camera models in the commercial market and it is very likely that different camera models with identical descriptors exist. It should be noted that uniqueness is not required to detect double-compressed videos in the proposed method. Details will be shown in Section 4.4

### 4.2. Descriptors of H.264 Hardware Video Encoder

In our experiments, 11 models of car DVR cameras (or black boxes) were used to evaluate the proposed method. Table 13 illustrates the basic parameters of H.264 encoder’s descriptors. There were baseline, main, and high profiles in car DVR cameras. The resolution of the H.264 videos with the baseline profile was 1280×720, and the other resolutions’ profile was 1920×1080.

Table 14 shows intra prediction modes in H.264 encoder’s descriptors. Models *B*, *D*, *E*, and *F* support all 16×16 intra prediction modes, and the rest of the models support only three without the planar mode (mode 3). Since the planar prediction mode is relatively complex compared to the others, it is expected that some hardware manufacturers did not implement planar prediction. For 4×4 intra prediction, there were several types of implementations. The first type does not support 4×4 intra prediction. The second one is to implement only the basic and important prediction modes: DC, horizontal, and vertical intra prediction. The last one is to support all intra prediction modes.

Table 15 shows inter block partitions in H.264 encoder’s descriptors. There are four types of inter block partition implementations in the car DVR models used in the experiments. The first type supports only 16×16 blocks, which is a minimum requirement to support inter block coding. The second type is to support 16×16 and 8×8 blocks, which are square blocks. The video encoders of the third type consider 16×16, 16×8, 8×16, and 8×8 blocks. The last one supports all inter block partitions defined in H.264.

The above experiments show that since H.264 hardware video encoders used in our experiments support a subset of encoding modes rather than the full set of encoding modes, the subset of encoding modes can be used as descriptors of H.264 video encoders. On the other hand, these H.264 descriptors are not completely unique to each other, unlike the HEVC descriptors. Models B, C, F, G, H, I, and K were unique to each other. The descriptors of models A and J were identical. Models D and E also have the same descriptors. Again, uniqueness is not required to detect double-compressed videos in the proposed method.

### 4.3. Descriptors of Double-Compressed Video Using Software Video Encoder

**Figure 5:**
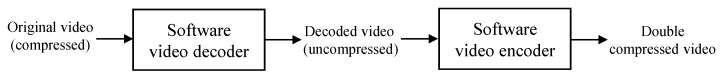
Validation method of the proposed method.

Figure 1 depicts the procedure of video forgery by double compression. In order to validate the proposed algorithm, the procedure of Figure 1 was modified to simple double compression in Figure 5. The process of editing in Figure 1 is skipped in Figure 5. The decoded video (uncompressed) from the software video decoder is directly fed to the software video encoder. Then, the descriptor of the double-compressed video in Figure 5 is extracted and compared with the descriptor of the original video (compressed).

After going through the procedure in Figure 5, the descriptor of a double-compressed video should be changed to a descriptor corresponding to the software video encoder, and the descriptor of the double-compressed video should be unrelated to the descriptor of the original video (compressed). To confirm the above, we conducted a simulation using JM software [35] for H.264 as the software video decoder and encoder in Figure 5. Since JM software is essentially a test model for H.264, the JM software video encoder can fully utilize the full set of encoding modes. We confirmed that the descriptors of the double-compressed video also utilize the full set of encoding modes in H.264. Since the decoded video (uncompressed) is simply raw video, it makes sense that the descriptor of the double-compressed video uses the full set of encoding options. No car DVR model used in our experiments utilizes the full set of encoding modes. Consequently, there was no car DVR model in which the descriptor of the original video (compressed) is identical to the descriptor of the double-compressed video.

Next, we conducted another simulation using an authoring tool (FFmpeg [33]). It is confirmed that the descriptors of a double-compressed video are unrelated to the descriptor of the original video (compressed). The descriptors of a double-compressed video are always changed to a descriptor corresponding to the authoring tool from whatever the descriptor of the original video (compressed) was. On the other hand, this authoring tool supports the full set of encoding modes except inter block partitions of 8×4, 4×8, and 4×4 blocks. Hence, the descriptor of this authoring tool is the same as the descriptor of Model *B* in Table 13. Here, we did not consider parameters in SPS and PPS, such as profile, level, GOP, etc.

In the same way, the experiments were performed on HEVC camera models using two software video encoders. One is HM software [36], which is a test model of HEVC. HM software was used for the software video decoder and encoder in Figure 5. Since HM software is a test model for HEVC, the descriptor from the HM software video encoder also utilized the full set of encoding modes. Next, we used the same authoring tool as above for the HEVC software video encoder by changing the configuration of the authoring tool to the HEVC codec. The descriptor of the double-compressed video was always changed to that of the software video encoder, whatever the descriptor of the original video (compressed) was. The authoring tool, unlike the HM software, did not support the full set of encoding modes. The maximum CU size of the authoring tool was 64×64, while the maximum CU size of HEVC camera models was 32×32. However, only inter blocks and skip blocks are supported except for intra block in 64×64 CU. All intra, inter, and skip blocks are allowed in 32×32, 16×16, and 8×8 CUs. Further, only inter block partitions of 2N×2N size are allowed, regardless of CU sizes. A total of 35 types of intra predictions are fully utilized in 2N×2N and N×N regardless of CU sizes. There was no camera model where the descriptor of the original video (compressed) are identical to the descriptor of the double-compressed videos using the HM software or authoring tool.

### 4.4. Video Forgery Detection

The proposed method does not replace the existing methods but complements them. For example, a target video is first examined using the proposed method. If the target video is determined as ‘*Not detectable*’, the existing methods are applied to further examine the target video. Since the proposed method is complementary, it does not matter whether the proposed method is superior to the existing methods or not. Therefore, only the experimental results of the proposed double compression detection are presented in this paper. In practice, the performance of the proposed method is highly dependent on the combination of encoders used for the first and second compressions.

Table 16 shows the experimental results of the proposed double compression detection based on Figure 4. It is assumed here that only two software video encoders are available for tampering videos, and two descriptors are registered in the model database. An unforged video (or original video) is captured using each camera model. The running time of each unforged video is about 30 s. Then, two forged videos are generated using two software video encoders: open-source and HM (or JM) test models. In our experiments, only double compression is performed without editing the original video, as shown in Figure 5. The total number of HEVC camera models used in the experiments is 13. The proposed method successfully decides unforged videos as unforged in all camera models. Further, forged video inputs are judged as forged, regardless of the camera model that the video is captured by. The accuracy of detection results is 100%. The reason is that the descriptors of the open-source and HM software for HEVC are different from the descriptors of all hardware video encoders used in the experiments, as described in Section 4.3. On the other hand, one descriptor of camera model *b* is identical to the descriptor of the open-source authoring tool based on H.264. Hence, the proposed method cannot judge whether the test video from camera model *b* is tampered with or not; the test video is categorized as ‘*Not detectable*’. The remaining videos from ten camera models are correctly detected. The accuracy of the detection results for the ten detectable camera models is 100%. The proposed method basically checks whether the descriptor of the original video (compressed) is changed to the new one or not. It does not matter for detecting double compression that two descriptors from different camera models are identical.

Now, let us consider a situation of tampering with a test video using the new software video encoder ($D_{S}^{new}$) that is not registered in the model database. If the proposed method decides the test video is forged, $D_{T}⊈D_{b}$ should be satisfied. This means that the descriptor of the test video is changed to the new one, and the test video is double-compressed. Hence, the proposed method provides the correct result in this case. Meanwhile, when the proposed method judges the test video as unforged, the decision result could be wrong if $D_{S}^{new}\subset D_{b}$ and all $D_{S}^{i}⊈D_{T}$. Although the test video is double-compressed, the descriptor of the double-compressed video is still included in $D_{b}$ in this case. The condition of all $D_{S}^{i}⊈D_{T}$ denotes that none of the descriptors the of software video encoder in the model database are included in the descriptor of the test video or are identical. Then, the proposed method will decide the double-compressed video as not *Not detectable* but *unforged*. However, we believe that this case is very rare for the following reason. Since the open-source encoder is free and public, it is very likely that the new software ($D_{S}^{new}$) will support at least all of the encoding modes supported by the open-source encoder ($D_{O}$), such as FFmpeg [33]. Then, $D_{O}\subset D_{S}^{new}$ is satisfied. Since $D_{O}$ is registered in the model database, the condition of all $D_{S}^{i}⊈D_{T}$ will be violated. In other words, $D_{S}^{new}$ should not support some encoding modes of the open-source encoder in order for the proposed method to make the wrong decision. However, it is not common that the performance of the new software video encoder provides worse performance than the open software encoder that is easily found for free. Note here that when the proposed method decides the test video is double compressed, the result is always correct.

## 5. Conclusions

In this paper, we present a new approach to detecting double-compressed videos using the proposed descriptor that represents the characteristics of each video encoder. Unlike human fingerprints, each video encoder’s descriptor is not completely unique enough to discriminate all video encoders. Nevertheless, we experimentally show that there are many types of descriptors according to video encoders. Specifically, the descriptors of hardware video encoders are different from those of software video encoders in most cases. Therefore, the proposed descriptor can be utilized to detect double-compressed video like human fingerprints.

The accuracy of the detection results is very important in digital forensics. Specifically, the untampered video should not be decided as a forgery video in order for the innocent not to be punished. The proposed method guarantees that the test video judged to be a forgery is indeed double compressed. In other words, the proposed method never determines a single-compressed video as a double-compressed video. We believe that this work is very meaningful for digital forensics. If an attacker is a video coding expert, one can easily find a way to neutralize the proposed method. For example, by modifying the test models such as HM or JM software, a software video encoder whose descriptor is identical to a specific hardware video encoder can be developed.

The proposed descriptor only includes whether a particular encoding mode is used. Not only whether the encoding mode is used but also the probability that the encoding mode is selected may be utilized in a descriptor of the video encoder. For example, according to our experiments, some hardware video encoders have a very high selection probability of modes 0, 1, and 2 among the 35 modes in HEVC intra prediction, but other encoders do not. The probability distribution of the chosen encoding modes is also an important characteristic of video encoders. We expect that considering the probability distribution will further improve our study.

## Figures and Tables

**Table 1:** Example of the H.264 decoder’s descriptor. BL stands for baseline.

Encoding Mode		Number
Profile		66(BL)
Level		3.1
GOP size		6
	mode0	385,332
Intra	mode1	30,440
16×16	mode2	95,570
	mode3	0
	mode0	690,994
	mode1	950,632
	mode2	1,476,582
Intra	mode3	0
4×4	mode4	0
	mode5	0
	mode6	0
	mode7	0
	mode8	0
	16×16	1,935,837
	16×8	0
Inter block	8×16	0
partition	8×8	1,171,112
	4×8	0
	8×4	0
	4×4	0

**Table 2:** Example of CU sizes and types in HEVC encoder’s descriptor.

CU Type	CU Size			
	64×64	32×32	16×16	8×8
Skip CU	11,609	48,399	35,748	28,864
Intra CU	0	35,040	35,838	26,458
Inter CU	16,097	32,277	14,679	6978

**Table 3:** Example of intra 2N×2N prediction modes of 32×32 CU in HEVC encoder’s descriptor. Here, IPMN stands for intra prediction mode number.

IPMN	Number	IPMN	Number	IPMN	Number	IPMN	Number
0	12,864	9	0	18	198	27	0
1	49,047	10	2908	19	0	28	0
2	1550	11	0	20	0	29	0
3	0	12	0	21	0	30	0
4	0	13	0	22	0	31	0
5	0	14	0	23	0	32	0
6	0	15	0	24	0	33	0
7	0	16	0	25	0	34	519
8	0	17	0	26	1854		

**Table 4:** Example of block partitioning information of 32×32 inter coded CU in HEVC encoder’s descriptor.

Size	Number	Size	Number
32×32	1,535,249	16×16	0
32×16	0	16×32	0
32×8	0	32×24	0
8×32	0	24×32	0

**Table 5:** Example of descriptor comparison. $N_{1}$, $N_{2}$, and $N_{3}$ are the number of times that each mode is used in Videos #1, #2, and #3.

Mode	Video #1	Video #2	Video #3
$M_{0}$	142,342	51,244	15,249
$M_{1}$	522,342	123,142	292,342
$M_{2}$	152,342	24,922	0
$M_{3}$	0	0	125,242
$M_{4}$	0	0	8754

**Table 6:** Example of 4×4 intra prediction in H.264. Here, descriptors from two videos shot by the same camera are identical to each other. Here, O and × represent ‘used’ and ‘not used’, respectively.

Encoding Mode	Video DB				Model DB
(4×4 Intra Pred.)	Video #1	Used	Video #2	Used	
mode0	722,157	O	1,095,886	O	O
mode1	747,453	O	1,490,516	O	O
mode2	1,324,614	O	1,790,590	O	O
mode3	0	×	0	×	×
mode4	0	×	0	×	×
mode5	0	×	0	×	×
mode6	0	×	0	×	×
mode7	0	×	0	×	×
mode8	0	×	0	×	×

**Table 7:** Example of 2N×2N intra prediction in 32×32 CU in HEVC. Here, descriptors from two videos shot by the same camera are not identical to each other. Here, O and × represent ‘used’ and ‘not used’, respectively.

Encoding Mode	Video DB				Model DB
(2N×2N Intra Pred.)	Video #1	Used	Video #2	Used	
mode0	3210	O	25,622	O	O
mode1	2702	O	31,559	O	O
mode2	54	O	1205	O	O
mode3	2	O	399	O	O
mode4	0	×	14	O	O
mode5	1	O	21	O	O
mode6	0	×	183	O	O
…	…	…	…	…	…
mode34	0	×	48	O	O

**Table 8:** Three types of comparison results. Here, O and × represent ‘used’ and ‘not used’, respectively.

Encoding Mode	Model DB	Test Video		
	*D*_*b*_	$D_{T}^{1}$	$D_{T}^{2}$	$D_{T}^{3}$
A	O	O	O	O
B	O	O	O	O
C	O	O	×	×
D	×	×	×	O
D	×	×	×	×
F	O	O	×	O
G	O	O	O	×

**Table 9:** One example of peculiar results in some H.264 hardware video encoders.

Model	16×16 Intra Prediction Modes							
	Mode 0		Mode 1		Mode 2		Mode 3	
	Num	%	Num	%	Num	%	Num	%
a	122,842	49.184	23,182	9.286	103,715	41.526	10	0.004
b	262,525	66.623	61987	15.731	69535	17.646	1	0.000

**Table 10:** Example of intra 2N×2N prediction modes of 32×32, 16×16, and 8×8 CUs in HEVC encoder’s descriptor. CM stands for camera model. Here, O and × represent ‘used’ and ‘not used’, respectively.

CM	CU Size	Intra 2N×2N Prediction Modes														
		0	1	2	3	4	5	6	7	8	9	10	11	…	33	34
1	32×32	O	O	O	×	×	×	×	×	×	×	O	×	…	×	O
	16×16	O	O	O	×	O	×	O	×	O	×	O	×	…	×	O
	8×8	O	O	O	O	O	O	O	O	O	O	O	O	…	O	O
2	32×32	O	O	O	×	×	×	×	×	×	×	O	×	…	×	×
	16×16	O	O	O	O	O	O	O	O	O	O	O	O	…	O	O
	8×8	O	O	O	O	O	O	O	O	O	O	O	O	…	O	O
3	32×32	O	O	×	×	×	×	×	×	×	×	O	×	…	×	O
	16×16	O	O	O	O	O	O	O	O	O	O	O	O	…	O	O
	8×8	O	O	O	O	O	O	O	O	O	O	O	O	…	O	O
4	32×32	O	O	O	O	O	O	O	O	O	O	O	O	…	×	O
	16×16	O	O	O	O	O	O	O	O	O	O	O	O	…	×	O
	8×8	O	O	O	O	O	O	O	O	O	O	O	O	…	×	O
5	32×32	O	O	O	O	O	O	O	O	O	O	O	O	…	O	O
	16×16	O	O	O	O	O	O	O	O	O	O	O	O	…	O	O
	8×8	O	O	O	O	O	O	O	O	O	O	O	O	…	O	O
6	32×32	O	O	O	O	O	O	O	O	O	O	O	O	…	O	O
	16×16	O	O	O	O	O	O	O	O	O	O	O	O	…	O	O

**Table 11:** Example of intra N×N prediction modes of 8×8 CU in HEVC encoder’s descriptors. Here, O and × represent ‘used’ and ‘not used’, respectively.

Camera	Intra N×N Prediction Modes													
Model	0	1	2	3	4	5	6	7	8	9	10	…	33	34
*a*	×	×	×	×	×	×	×	×	×	×	×	…	×	×
*b*	O	O	×	O	×	O	×	O	×	O	×	…	O	×
*c*	O	O	O	O	O	O	O	O	O	O	O	…	×	O
*d*	O	O	O	O	O	O	O	O	O	O	O	…	O	×
*e*	O	O	O	O	O	O	O	O	O	O	O	…	O	O

**Table 12:** Example of block partitioning of inter coded CU in HEVC encoder’s descriptors. Here, inter predict modes 0, 1, 2, 3, 4, 5, 6, and 7 correspond to 2N×2N, N×N, 2N×N, N×2N, 2N×nU, 2N×nD, nL×2N, and uR×2N PUs, respectively. Here, O and × represent ‘used’ and ‘not used’, respectively.

Camera	CU	Type of PU							
Model	Size	0	1	2	3	4	5	6	7
	32×32	O	×	×	×	×	×	×	×
*A*	16×16	O	×	×	×	×	×	×	×
	8×8	O	×	×	×	×	×	×	×
	32×32	O	O	O	×	×	×	×	×
*B*	16×16	O	O	O	×	×	×	×	×
	8×8	O	×	×	×	×	×	×	×
	32×32	O	O	O	×	O	O	O	O
*C*	16×16	O	O	O	×	O	O	O	O
	8×8	O	O	O	×	×	×	×	×
*D*	32×32	O	O	O	×	×	×	×	×
	16×16	O	O	O	O	×	×	×	×

**Table 13:** Examples of basic parameters in H.264 encoder’s descriptors.

Model	Profile	Level	Width	Height
*A*	Baseline	3	1280	720
*B*	High	4.1	1920	1080
*C*	High	4	1920	1080
*D*	High	4.2	1920	1080
*E*	High	4.2	1920	1080
*F*	High	4.2	1920	1080
*G*	High	4.1	1920	1080
*H*	High	4	1920	1080
*I*	Main	4	1920	1080
*J*	Baseline	3	1280	720
*K*	Baseline	3.1	1280	720

**Table 14:** Examples of intra prediction modes in H.264 encoder’s descriptors. Here, O and × represent ‘used’ and ‘not used’, respectively.

Model	Intra 16×16				Intra 4×4								
	0	1	2	3	0	1	2	3	4	5	6	7	8
*A*	O	O	O	×	×	×	×	×	×	×	×	×	×
*B*	O	O	O	O	O	O	O	O	O	O	O	O	O
*C*	O	O	O	×	O	O	O	×	×	×	×	×	×
*D*	O	O	O	O	×	×	×	×	×	×	×	×	×
*E*	O	O	O	O	×	×	×	×	×	×	×	×	×
*F*	O	O	O	O	×	×	×	×	×	×	×	×	×
*G*	O	O	O	×	×	×	×	×	×	×	×	×	×
*H*	O	O	O	×	O	O	O	O	O	O	O	O	O
*I*	O	O	O	×	O	O	O	×	×	×	×	×	×
*J*	O	O	O	×	×	×	×	×	×	×	×	×	×
*K*	O	O	O	×	O	O	O	O	O	O	O	O	O

**Table 15:** Examples of inter block partitions in H.264 encoder’s descriptors. Here, O and × represent ‘used’ and ‘not used’, respectively. The numbers 0, 1, 2, 3, 4, 5, and 6 in inter block partitions denote 16×16, 16×8, 8×16, 8×8, 8×4, 4×8, and 4×4, respectively.

Model	Inter Block Partition						
	0	1	2	3	4	5	6
*A*	O	O	O	O	×	×	×
*B*	O	O	O	O	×	×	×
*C*	O	×	×	O	×	×	×
*D*	O	×	×	×	×	×	×
*E*	O	×	×	×	×	×	×
*F*	O	O	O	O	O	O	O
*G*	O	O	O	O	×	×	×
*H*	O	O	O	O	×	×	×
*I*	O	×	×	O	×	×	×
*J*	O	O	O	O	×	×	×
*K*	O	O	O	O	×	×	×

**Table 16:** Results of the proposed double compression detection. Here, ‘Accuracy’ refers to the accuracy of detection results for detectable videos, not including undetectable videos.

Input	# of Camera Models	Detectable	Not Detectable	Accuracy (%)
Unforged Video (HEVC)		13	0	100
Forged Video (HEVC) using open-source	13	13	0	100
Forged Video (HEVC) using HM source		13	0	100
Unforged Video (H.264)		10	**1**	100
Forged Video (H.264) using open-source	11	10	**1**	100
Forged Video (H.264) using JM source		10	**1**	100

## Data Availability

All data derived from this study are presented in the article.
